# Total and Carboxylated Osteocalcin Associate with Insulin Levels in Young Adults Born with Normal or Very Low Birth Weight

**DOI:** 10.1371/journal.pone.0063036

**Published:** 2013-05-03

**Authors:** Päivi M. Paldánius, Kaisa K. Ivaska, Petteri Hovi, Sture Andersson, Johan G. Eriksson, Kalervo Väänänen, Eero Kajantie, Outi Mäkitie

**Affiliations:** 1 Children’s Hospital, Helsinki University Central Hospital, and Institute of Clinical Medicine, University of Helsinki, Helsinki, Finland; 2 Novartis Pharma AG, Basel, Switzerland; 3 Department of Cell Biology and Anatomy, Institute of Biomedicine, University of Turku, Turku, Finland; 4 National Institute for Health and Welfare, Helsinki, Finland; 5 Department of General Practice and Primary Health Care, University of Helsinki, Helsinki, Finland; 6 Helsinki University Central Hospital, Unit of General Practice, Helsinki, Finland; 7 Folkhälsan Research Centre, Helsinki, Finland; Broad Institute of Harvard and MIT, United States of America

## Abstract

**Objective:**

Osteocalcin (OC), a bone-derived protein, has been implicated in the regulation of glucose and energy metabolism. Young adults born with very low birth weight (VLBW) have altered glucose regulation and lower bone mineral density (BMD) compared with those born at term. The aim of this study was to explore the association between bone and glucose metabolism in healthy young adults born prematurely or at term.

**Methods:**

The cohort of this cross-sectional study comprised 332 non-diabetic young adults (age 18 to 27 years) born either preterm with VLBW (n = 163) or at term (n = 169). OC, carboxylated osteocalcin (cOC) and markers of glucose metabolism were measured at fasting and after a 75-g oral glucose tolerance test (OGTT).

**Results:**

VLBW adults were shorter, had lower BMD (p<0.001) and higher fasting OC (p = 0.027) and cOC (p = 0.005) than term-born subjects. They also had higher 2-hour insulin (p = 0.001) and glucose (p = 0.037) concentrations. OGTT induced a significant reduction in OC (p<0.001), similar in both groups. OC reduction was not associated with OGTT-induced increases in insulin (p = 0.54). However, fasting total OC and cOC correlated negatively with fasting insulin after adjustment for age, gender, BMD and VLBW status (r = −0.182, p = 0.009 and r = −0.283, p<0.001, respectively).

**Conclusion:**

Adults born with VLBW have higher OC and cOC than their peers born at term. This may in part reflect the mechanisms that underlie their lower BMD and decreased insulin sensitivity. Serum OC appears to be negatively associated with long-term glucose regulation whereas acute changes during OGTT may be mediated via other mechanisms.

## Introduction

Glucose homeostasis depends on a complex signal network orchestrated by the pancreatic islet cells, liver, fat, muscle, kidney and brain. The role of the skeleton in glucose and energy homeostasis has recently gained much attention [1], [2], [3]. The osteoblast-specific protein osteocalcin (OC) has been recognized as an endocrine factor with a proposed role in the regulation of glucose and energy metabolism by influencing insulin secretion and insulin sensitivity [4], [5], [6]. Vitamin K –dependent post-translational γ-carboxylation of OC leads to formation of carboxylated OC whereas only the uncarboxylated form of OC has been indicated in some studies to induce expression of adiponectin, insulin, and markers of pancreatic islet cell proliferation [5].

Preliminary data indicate that the cross-talk between bone and glucose-insulin metabolism could in part be mediated by OC [7]. Presence of such regulatory pathways in humans has not yet been confirmed [8], even if the body of knowledge is constantly increasing [9]. An inverse association between OC and markers of metabolic dysfunction has been reported in clinical studies evaluating markers of impaired glucose regulation [10], [11], [12], [13], [14]. While some of the reported associations are relatively strong, no direct causal relationship between changes in glucose regulation, insulin sensitivity and changes in OC has been confirmed in humans. The published studies include mostly cross-sectional *post hoc* analyses of elderly populations with confounding factors related to high age and co-morbidities such as type 2 diabetes, impaired glucose tolerance or dyslipidemia, which complicate analysis and interpretation of results [15], [16], [17]. However, the limited human data regarding the role of OC carboxylation are inconclusive [9].

Young adults born preterm with very low birth weight (VLBW), while mostly normoglycemic, have lower insulin sensitivity when compared with those born at term [18], [19]. They also have lower bone mineral density (BMD) than their peers born at term, when studied close to the age of peak bone mass attainment [20]. The developmental period that differs most dramatically between the VLBW and term-borns occurs after VLBW birth, during the period that would normally be the third trimester of pregnancy. This period is important for the development and adjustment of endocrine and metabolic systems, probably through metabolic programming, even if the exact mechanisms are unknown [21], [22], [23]. This time is also crucial for fetal bone mineralization as up to 80% of the body calcium of a term newborn is being accrued during the last trimester [24]. The alterations in skeletal health and glucose tolerance observed in VLBW subjects in early adulthood provide an opportunity to further study the regulatory pathways between glucose, insulin and bone metabolism.

The aim of this study was to explore the association between bone and glucose metabolism by evaluating OC, carboxylated osteocalcin (cOC) and markers of glucose and insulin metabolism before and during a standard oral glucose tolerance test in apparently healthy young adults born either prematurely with VLBW or at term.

## Materials and Methods

### Ethics Statement

The Helsinki and Uusimaa Hospital District Ethics committee approved the study protocol and a written informed consent was given by all study participants. The study was carried out according to the principles of Declaration of Helsinki.

### Subjects

The original study cohort comprised 335 consecutive prematurely born (gestational age <37 weeks) VLBW infants born between January 1978 and December 1985 who were discharged alive from the neonatal intensive care unit of Children’s Hospital at Helsinki University Central Hospital, Finland. A comparison group was selected from the records of all consecutive births at each birth hospital. For each VLBW survivor, the next available singleton infant born at term (gestational age ≥37 weeks) of the same sex who was not small for gestational age (standard-deviation score for birth weight ≥−2.0) was selected. The subjects were traced in young adulthood in 2004 through data from the Population Register Centre of Finland. Mortality from hospital discharge to June 2004 was 1.8% for the VLBW subjects and 1.0% for the comparison group born at term. Birth weight ranged from 600 to 1500 g in the VLBW group and from 2560 to 4930 g in the term group; gestational age ranged from 24.0 to 35.6 weeks in the VLBW group and from 37.0 to 42.9 weeks in the term group.

Among the survivors, 95.1% of VLBW subjects and 96.8% of subjects born at term were identified at young adulthood. A total of 255 VLBW subjects and 314 subjects born at term who were living in the greater Helsinki area were invited to participate in the study. A total of 338 subjects agreed to participate; 166 of the VLBW subjects (65.1%) and 172 of the subjects born at term (54.8%). Subjects with type 1 diabetes (n = 1), concomitant or chronic systemic glucocorticoid use (n = 1), pregnancy (n = 2) or insufficient fasting prior to OGTT (n = 2) were excluded from the analyses. After exclusions altogether 332 subjects were eligible for the study (Table 1).

**Table 1:** Characteristics of the study population (n = 332).

		Term	VLBW	p value
Subjects, n		169	163	
Gender, M/F		68/101	71/92	0.54
Gestational age, weeks		40.1 (1.2)	29.2 (2.2)	**<0.001**
Birth weight, g		3585.7 (467.7)	1122.3 (220.5)	**<0.001**
Age at study assessment, years		22.5 (2.2)	22.4 (2.1)	0.93
Height, cm	M	180.5 (6.4)	174.6 (7.7)	**<0.001**
	F	167.2 (6.8)	162.0 (7.6)	**<0.001**
Weight, kg	M	76.1 (67.2)	67.2 (13.1)	**<0.001**
	F	63.5 (10.8)	58.5 (12.0)	**0.002**
BMI, kg/m^2^	M	23.3 (3.2)	22.0 (3.6)	**0.022**
	F	22.7 (3.7)	22.3 (3.9)	0.41
Lumbar spine aBMD,Z-score		−0.42 (1.05)	−0.94 (0.98)	**<0.001**
Leisure time exercise frequency, n (%)				0.084
None		4 (2.4)	11 (6.7)	
Less than once per month		19 (11.2)	12 (12.9)	
Once or twice a month		16 (9.5)	22 (13.5)	
Once a week		33 (19.5)	31 (19.0)	
2 to 3 times a week		63 (37.3)	42 (25.8)	
4 to 5 times a week		18 (10.7)	17 (10.4)	
Daily		16 (9.5)	15 (9.2)	
Not known		0 (0)	4 (2.5)	
Education level of more educated parent at least university degree		48.2%	36.2%	0.067

Values are mean (SD) and the p values are for the difference between Term and VLBW groups with one-way ANOVA or Pearson’s chi-square test. Significant p values are shown in bold. DXA results were available for 284/332 participants.

### Clinical Characteristics

All 332 subjects were invited to attend the clinic at the National Public Health Institute after an overnight fast of at least 10 hours. For five subjects the overnight fasting period was 7–10 hours but they were included in the analysis. The subjects were evaluated for anthropometry (weight, height, body mass index; BMI), medical history (including self-reported parents’ history of type 1 and type 2 diabetes), leisure-time physical activity and socioeconomic status, as previously described [18], [20].

### Oral Glucose Tolerance Test (OGTT)

A 2-hour OGTT (75 g glucose) was initiated between 6∶13 am and 11∶11 am. Plasma and serum samples were collected at baseline (0 min) and at 120 min according to standard protocols. Plasma glucose concentrations were measured at 0 min and 120 min by spectrophotometric hexokinase and glucose-6-phosphate dehydrogenase assay (Gluko-quant glucose/hexokinase, Roche Diagnostics) with a Hitachi Modular automatic analyzer. At glucose concentration of 4.7 mmol per liter (84.7 mg per deciliter), the inter-assay coefficient of variation is 2.3% [25]. Impaired glucose tolerance was defined according to WHO as plasma glucose concentration >6.1 but ≤7.0 mmol/l at 0 min and/or ≥7.8 mmol/l but <11.1 mmol/l at 120 min. Diabetes was defined as plasma glucose concentration ≥7.0 mmol/l at 0 min and/or ≥11.1 mmol/l at 120 min. Based on OGTT findings, all subjects were considered normoglycemic. Serum samples were stored at −70°C until further analysis.

### Measurement of Insulin and HOMA-IR

Serum insulin was measured with time-resolved immunofluorometric assay (Perkin Elmer Life Sciences, Finland) with a detection limit of 0.5 mU per liter (3 pmol per liter) and an interassay coefficient of variation of less than 4%. The insulin-resistance index determined by homeostasis model assessment (HOMA-IR) was calculated as the product of the fasting serum insulin concentration (in milliunits per liter) and fasting plasma glucose concentration (in millimoles per liter) divided by 22.5 [26].

### Serum Osteocalcin

Serum total OC and serum γ-carboxylated osteocalcin (cOC) were determined at 0 min and 120 min by previously described two-site immunoassay protocols [27]. Two site immunoassay for serum total OC is based on monoclonal antibodies (Mabs) 2H9 and 6F9 and detects the N-terminal midsegment of the OC molecule. Assay for carboxylated OC (cOC; Mabs 6F9 and 3H8) detects the same fragments but prefers γ-carboxyglutamic acid (Gla) containing forms of OC, with <10% cross-reactivity to fully uncarboxylated OC (ucOC) [28]. Briefly, 200 ng of biotinylated capture Mab and 100 ng of europium-labelled tracer Mab per well was used. Synthetic peptide of human OC amino acids 1–49 (Advanced Chemtech, USA) was used as a calibrator. Streptavidin-coated microtiter plates were from Kaivogen (Turku, Finland) and other immunoassay reagents (Delfia® Assay Buffer, Wash Solution and Enhacement solution) from Perkin Elmer Life Sciences (Turku, Finland). Time-resolved fluorescence was measured with Victor2 Multilabel Counter (PerkinElmer Life Sciences). All samples were measured as duplicates and simultaneously at the end of the study. In order to reduce the bias by inter-assay variability, samples for each subject (0 min and 120 min) were analyzed in parallel. The intra- and inter-assay variations were 4.4% and 9.0%, for total OC and 2.5% and 8.0% for cOC, respectively. Due to lack of validated method for accurate analysis of ucOC values we used a calculated value for uncarboxylated OC, which was derived from total OC and cOC values (ucOC = total OC minus cOC).

### Bone Densitometry

Bone mineral content (BMC) and areal BMD (aBMD) for the lumbar spine (L1–L4) were measured with dual-energy X-ray absorptiometry (DXA, Hologic Discovery A, software version 12.3∶3) and transformed into age-adjusted Z scores using the equipment-, age-, and sex-specific reference data. A cut-off Z score value of −1.0 was chosen to define reduced BMD [29]. Body composition, including lean body mass and fat percent (fat-%), was determined with the same DXA equipment.

A total of 284 subjects (of 332) were available for the analysis of lumbar spine aBMD, which was chosen as the site for analysis due to its high content of trabecular bone and active turnover. The reasons for the missing BMD results included unwillingness to undergo DXA, pregnancy, cerebral palsy, several compressed lumbar vertebrae or foreign objects in the scanning area. Scans with foreign objects such as surgical fixation material or jewelry in the measurement area (five subjects) were omitted from the analysis. If more than one lumbar vertebra was compressed, the corresponding lumbar spine scan was excluded from analysis (two subjects); if only one lumbar vertebra was compressed, BMD without the affected vertebra was used (seven subjects).

### Statistical Analysis

Glucose, insulin and osteocalcin concentrations and HOMA-IR were non-normally distributed (Shapiro-Wilk test <0.95) and were used after logarithmic transformation. The difference between Term and VLBW groups was analyzed with one-way ANOVA. Standardized linear regression coefficients (β_std_) between the analytes or the 120 min changes in the analytes were determined using linear regression. Multiple regression analysis was used to adjust for age, gender, VLBW status or BMD. We used SPSS for Windows 16.0 (SPSS Inc., Chicago, IL) for statistical analyses, except for linear regression which were calculated using Statistica for Windows 7.1 (StatSoft Inc., Tulsa, OK). P values <0.05 were considered statistically significant.

## Results

The cohort comprised 332 young Finnish adults born either with VLBW (n = 163; 92 females, 71 males) or at term (n = 169; 101 females, 68 males). The age of the subjects ranged from 18 to 27 years in both groups. There was no difference in age between the groups at the time of evaluation (mean age 22.4 vs. 22.5 years for VLBW subjects and those born at term, respectively, p = 0.93). The mean (± SD) birth weight in the VLBW group was 1122.7 g (217.6) and 3570.8 g (520.8) for subjects born at term. The mean adult weight, height and BMD were significantly lower in subjects born with VLBW than in those born at term.

Fasting glucose and insulin concentrations were within normal ranges in both groups but insulin levels were marginally higher in VLBW subjects (+11%, p = 0.068) when compared to those born at term. This difference between groups was also statistically significant between the female VLBW subjects and their term-born peers as the VLBW females had higher baseline insulin values than their term-born controls (5.8 vs. 5.0 mU/L, p = 0.045). There was no difference in fasting glucose levels between the groups (p = 0.24) or between the genders (Table 2). In contrast, at 120 min all subjects with VLBW had significantly higher insulin (+30.5%, p = 0.001) and glucose concentrations (+5.5%, p = 0.037) than those born at term (unadjusted) (Table 2) as previously reported [18].

**Table 2:** Serum levels of insulin, glucose, and osteocalcin before (fasting) and after (120 min) OGTT in the Term and VLBW groups.

			Term	VLBW	p value
Glucose (mmol/L)	Fasting	All	4.7 (1.1)	4.7 (1.1)	0.244
		M	4.9 (1.1)	4.9 (1.1)	0.874
		F	4.5 (1.1)	4.6 (1.1)	0.233
	120 min	All	5.1 (1.3)	5.3 (1.3)	**0.037**
		M	4.9 (1.3)	5.2 (1.2)	0.167
		F	5.2 (1.3)	5.5 (1,3)	0.097
	Δ120 min(%)		11.6%	16.1%	0.259
Insulin (mU/L)	Fasting	All	5.1 (1.7)	5.6 (1.7)	0.068
		M	5.2 (1.8)	5.4 (1.6)	0.671
		F	5.0 (1.7)	5.8 (1.7)	**0.045**
	120 min	All	26.2 (2.2)	34.1 (1.9)	**0.001**
		M	20.2 (2.6)	26.8 (1.8)	**0.032**
		F	31.2 (1.9)	41.1 (1.9)	**0.003**
	Δ120 min(%)		545%	625%	0.065
HOMA-IR	Fasting	All	1.0 (1.8)	1.2 (1.7)	0.060
		M	1.1 (1.9)	1.2 (1.6)	0.661
		F	1.0 (1.8)	1.2 (1.8)	**0.042**
Total osteocalcin(ng/mL)	Fasting	all	11.3 (1.5)	12.4 (1.5)	**0.027**
		M	14.0 (1.4)	14.6 (1.4)	0.468
		F	9.8 (1.5)	11.0 (1.4)	**0.030**
	120 min	all	9.1 (1.5)	10.2 (1.5)	**0.016**
		M	11.5 (1.3)	12.2 (1.4)	0.249
		F	7.8 (1.5)	8.9 (1.5)	**0.028**
	Δ120 min(%)		−18.1%	−16.7%	0.515
Carboxylatedosteocalcin (ng/mL)	Fasting	all	10.6 (1.5)	12.0 (1.5)	**0.005**
		M	12.9 (1.4)	14.2 (1.5)	0.117
		F	9.2 (1.5)	10.5 (1.5)	**0.021**
	120 min	all	8.5 (1.5)	9.6 (1.5)	**0.007**
		M	10.3 (1.4)	11.8 (1.5)	**0.043**
		F	7.4 (1.5)	8.3 (1.5)	0.061
	Δ120 min(%)		−18.8%	−18.2%	0.896

Values are geometric means (SD) and the p values are for the difference between Term and VLBW groups with one-way ANOVA (after logarithmic transformation) or Mann-Whitney nonparametric test (%-changes). Results are shown for the entire cohort (n = 331) and for males (n = 138) and females (n = 193) separately. Significant p values are shown in bold. HOMA-IR was calculated as Fasting Insulin * Fasting Glucose divided by 22.5.

**Figure 1:**
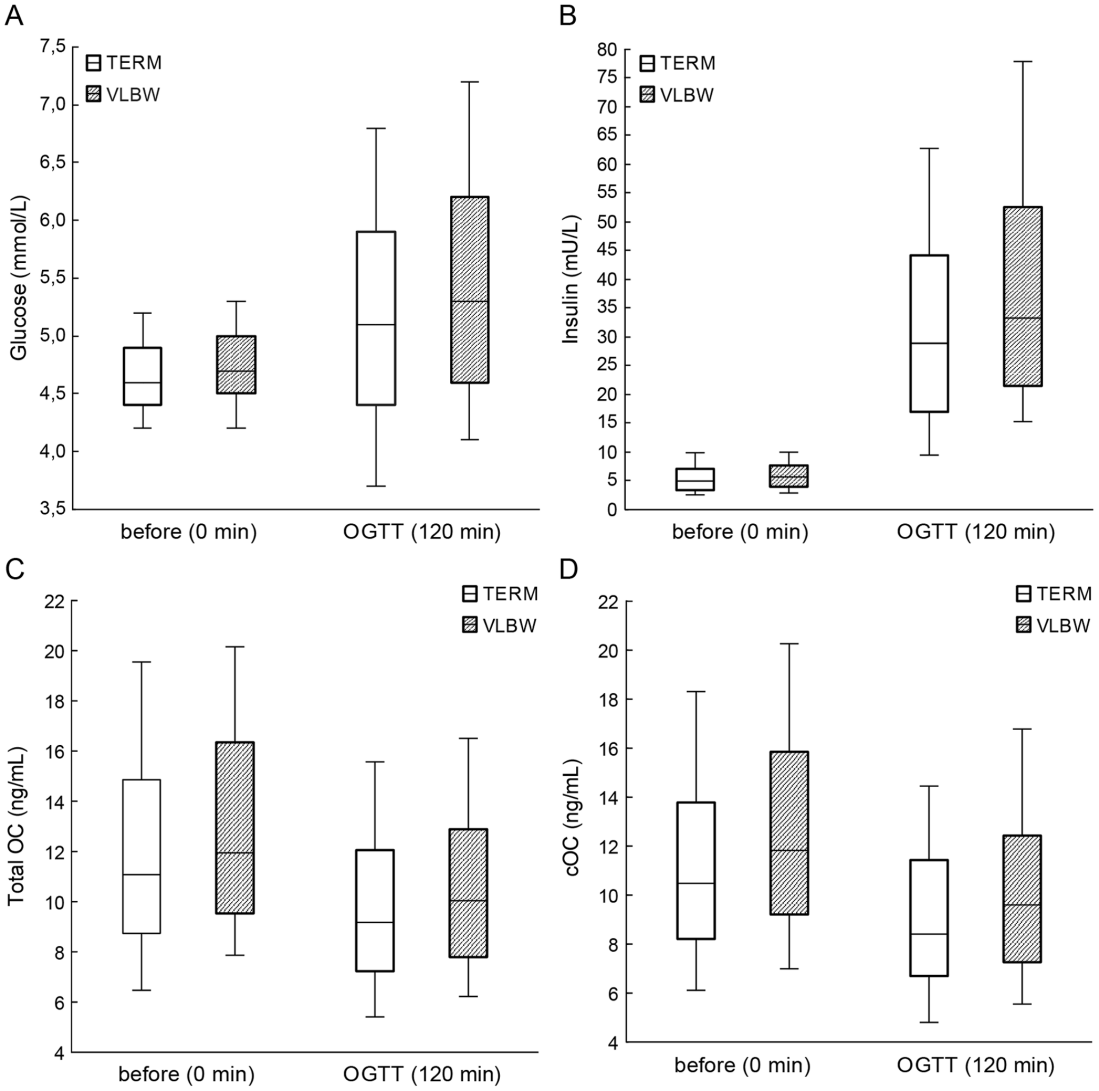
Glucose (A), insulin (B), total osteocalcin (C) and carboxylated osteocalcin (D) before and after 2-hour oral glucose tolerance test in term and VLBW subjects. The lines inside the boxes represent the 50th percentile; the limits of the boxes represent the 25th and 75th percentiles, and the whiskers represent the 10th and 90th percentiles.

Subjects with VLBW had higher fasting levels of OC (ANOVA, p = 0.027) and cOC (p = 0.005) than the term-born subjects. Total OC and cOC remained higher also after OGTT in VLBW subjects as compared with those born at term (ANOVA, p = 0.016 and p = 0.007, respectively) (Table 2, Figure 1). Male subjects had significantly higher levels of total OC and cOC than female subjects, both in the VLBW group and in subjects born at term (p<0.001 for all). The difference between subjects with VLBW and those born at term tended to be more pronounced in females but the difference did not reach statistical significance. The mean lumbar spine Z score values were significantly lower in VLBW subjects (−0.94) than in those born at term (−0.42, p<0.001).

**Figure 2:**
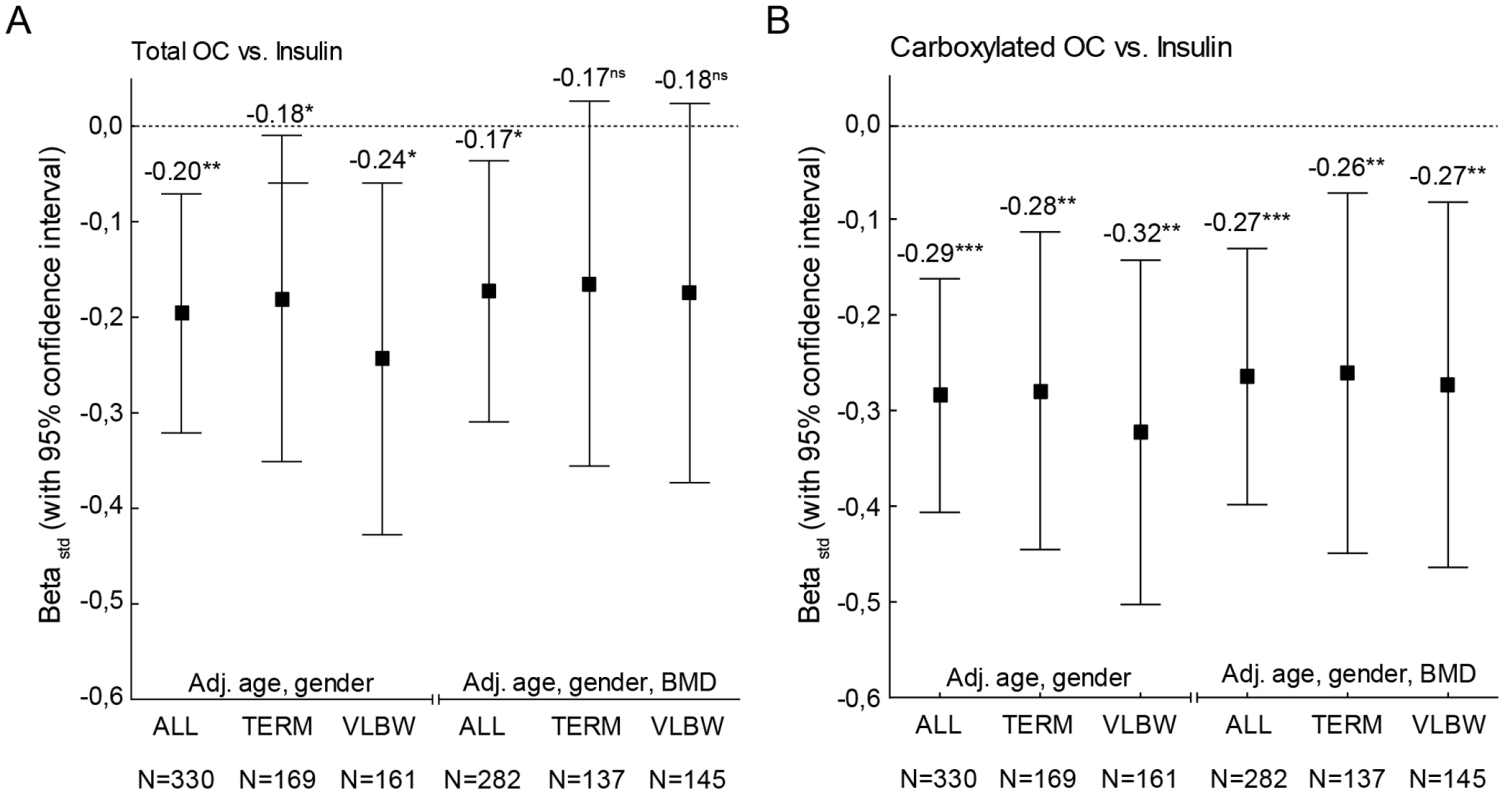
Standardized linear regression coefficients (βstd) between fasting insulin and A) total osteocalcin or B) carboxylated osteocalcin. Results are shown separately for the entire cohort (All) and the two groups (Term, VLBW) and they have been adjusted for age, gender and Z-score for lumbar spine BMD. Squares indicate the value for βstd, and the whiskers represent 95% confidence interval. The P values are indicated with asterisks: *, P<0.05; **, P<0.01; ***, P<0.001; ns, not significant.

Fasting total OC and cOC were negatively correlated with fasting insulin levels after adjustment for age, gender, VLBW status and BMD (r = −0.182, p = 0.009 and r = −0.283, p<0.001, respectively) (Table 3). The correlation between fasting insulin and cOC remained significant also after adjustment for lean body mass (r = −0.242, p<0.001) and whole body fat percent (r = −0.144, p = 0.028) (Table 3). The correlation between insulin and OC was similar in both groups, although the r -values were generally larger in the VLBW group (Figure 2). There was no significant association between insulin and total OC when the correlations were further adjusted for body fat percentage. As expected, total OC and lumbar spine Z-score were inversely correlated in all subjects (β_std_ = −0.239, p<0.001) independent of gender or VLBW status.

**Table 3:** Correlations between osteocalcin and parameters of glucose metabolism.

	Fasting (0 min) Insulin			120 min Glucose		
	*Beta_std_*	*+/*−*95% CI*	*p value*	*Beta_std_*	*+/*−*95% CI*	*p value*
**Total osteocalcin**						
Unadjusted	−0.103	−0.211, 0.005	0.063	**−0.127**	−0.235, −0.019	**0.021**
adj. age, gender, VLBW	**−0.214**	−0.340, −0.089	**<0.001**	−0.128	−0.258, 0.002	0.053
adj. age, gender, VLBW, lumbar spine BMD	**−0.182**	−0.319, −0.045	**0.009**	−0.099	−0.241, 0.042	0.168
adj. age, gender, VLBW, lean body mass	**−0.156**	−0.386, −0.031	**0.021**	−0.100	−0.144, 0.025	0.850
adj. age, gender, VLBW, fat-%	−0.107	−0.312, 0.025	0.096	−0.044	−0.111, 0.058	0.535
**Carboxylated osteocalcin**						
Unadjusted	**−0.169**	−0.276, −0.006	**0.002**	−**0.117**	−0.225, −0.009	**0.034**
adj. age, gender, VLBW	**−0.312**	−0.435, −0.190	**<0.001**	−0.117	−0.245, 0.010	0.071
adj. age, gender, VLBW, lumbar spine BMD	**−0.283**	−0.418, −0.149	**<0.001**	−0.090	−0.230, 0.049	0.202
adj. age, gender, VLBW, lean body mass	**−0.242**	−0.480, −0.143	**<0.001**	−0.086	−0.129, 0.030	0.224
adj. age, gender, VLBW, fat-%	**−0.144**	−0.350, −0.030	**0.028**	−0.006	−0.084, 0.077	0.938

Values are standardized linear regression coefficients (Beta_std_) with 95% confidence intervals (CI). Statistically significant values are **bolded**. Fasting osteocalcin values were used to evaluate association to fasting insulin and 2-hour osteocalcin values were used to study association to 2-hour glucose values.

The lack of validated methods for the accurate analysis of ucOC limited the analysis of the ucOC values to the use of theoretical estimates based on the total OC and cOC measurements. Fasting insulin levels correlated positively with calculated proportion of uncarboxylated OC (ucOC) (r = 0.176, p = 0.001) and this correlation was significant also after adjustment for age, gender and VLBW status (r = 0.193 p<0.001). The total OC correlated negatively and the calculated ucOC positively with fasting insulin when adjusted for age, gender, VLBW status and lean body mass (r = −0.156, p = 0.021 and r = 0.153, p = 0.007, respectively).

Elevated post-load glucose levels after OGTT is a validated marker of impaired glucose regulation and predicts progression to diabetes in subjects with normal fasting glucose [30]. Glucose concentrations at 120 min were moderately associated with total OC (p = 0.021) and cOC (p = 0.034) concentrations at 120 min. However, the associations between glucose and OC were not significant after any adjustments (Table 3).

Fasting insulin is a driver of homeostasis model assessment of insulin resistance index HOMA-IR. HOMA-IR was strongly correlated with OC and cOC at 0 min, independent of age, gender, VLBW status and height (p<0.001 for all correlations) and independent of lumbar spine BMD (r = 0.180, p = 0.010 and r = −0.277, p<0.001 for total OC and cOC, respectively). The association between HOMA-IR and total OC was no longer significant (p>0.05) when further adjusted for BMI, whereas association to cOC remained significant (r = −0.126, p = 0.038).

The acute glucose load during OGTT induced a significant reduction in total OC and cOC levels in all subjects, the mean (95% CI) decreases being −17.4% (−19.0, −15.8) and −18.5% (−20.0, −16.9) for OC and cOC, respectively. The reduction of OC and cOC values was similar in VLBW subjects and in controls (for OC p = 0.382, and for cOC p = 0.708) (Figure 1). In the entire cohort, the OGTT-induced reductions in OC values were not associated with simultaneous increases in insulin levels (p = 0.54) but were, however, weakly associated with OGTT-induced increase in glucose levels (r = −0.13, p = 0.022) (Table 4). Changes in glucose and insulin concentrations during OGTT correlated strongly with each other (r = 0.663, p<0.001, Table 4).

**Table 4:** Standardized linear regression coefficients (Beta_std_) between OGTT-induced changes (Δ120 min, from 0 to 120 min) in glucose, insulin and osteocalcin levels.

	Δ120 minInsulin	Δ120 minTotal OC	Δ120 mincOC
Δ120’ minGlucose	0.663 ***	−0.215 *	−0.053
Δ120 min Insulin		−0.034	−0.065
Δ120 min Total OC			0.753***

P values are indicated with asterisks (*p<0.05, ***p<0.001).

## Discussion

Our study shows that fasting insulin levels are associated with circulating OC values, particularly cOC, in young adults. All subjects were non-diabetic but VLBW subjects in this cohort have previously been reported to present with signs of impaired glucose regulation [18]. The association between OC and fasting insulin values was independent of age, gender and VLBW status, indicating that OC may participate in long-term regulation of glucose-insulin metabolism particularly in subjects with metabolic challenges, such as the studied population with VLBW.

Impaired glucose tolerance is an indicator of impaired regulation of glucose metabolism and reduced peripheral insulin sensitivity. Post-load OC and cOC values in our study correlated only weakly with glucose or insulin values at 120 min. Thus, we suggest that OC does not seem to be the main mediator of acute glucose regulation in humans during OGTT. Animal studies in mice suggest that insulin signalling in osteoblasts increases the secretion of OC and thereby promotes glucose homeostasis via uncarboxylated OC and may thus prevent the development of insulin resistance, glucose intolerance and abnormal weight gain [3]. Several mechanisms have been proposed, including stimulation of osteoblast differentiation and OC production, and increased release of uncarboxylated osteocalcin from the bone matrix due to increased bone resorption via alterations in RANK-RANKL-OPG pathway [6], [31]. It is unclear if similar regulatory system is present in humans [9], [32]. Furthermore, it is not known how rapid the suggested effect of insulin is and whether 2-hour OGTT is the best method to evaluate the effect. Our results do not, however, exclude the possibility that insulin-induced OC may regulate glucose and bone metabolism over a longer or shorter time period than the interval used in this study. Our data indicate that, in humans, OC and cOC levels rapidly and significantly decrease following the glucose load but the magnitude is not associated with increases in insulin levels.

Insulinotropic incretin hormones have been excluded as potential key mediators for the immediate reduction in bone formation after a meal in humans [33], [34]. In animal studies, however, glucose-dependent insulinotropic peptide (GIP) has been observed to both inhibit bone resorption and stimulate bone formation [35]. Thus, the OGTT-induced suppression of OC and cOC observed in our study is likely to be mediated via a different mechanism. In order to isolate the hormonal role of OC from its role as a marker of bone formation we previously conducted a pilot study in a subset of the cohort now studied [36], in which, we assessed the changes in the markers of bone formation and resorption together. We demonstrated that OGTT-induced changes in OC are associated with OGTT-induced changes in bone formation, but not resorption markers [36]. Regulation of glucose metabolism in response to rapidly applied glucose load might, however, differ from that observed after more physiological intake of nutrients.

A limited number of studies have evaluated bone metabolism in VLBW subjects. Some studies have reported increased OC in subjects born with VLBW [37] while in another study (which did not assess OC) no differences in formation or resorption markers were observed [19]. In our study, OC and cOC were higher in subjects born with VLBW and elevated OC levels could thus partially explain the decreased insulin sensitivity in VLBW subjects. This is somewhat contradictory to previous, mostly preclinical findings in mice on the role of OC in energy metabolism [4], [5], [6]. The baseline insulin and OC values were also higher in female VLBW subjects than in females born at term but there is no general, significant interaction between genders in this VLBW cohort [18]. Also our data indicate that the gender-specific differences are not significantly impacting the suggested role of OC in acute glucose regulation. Subjects with VLBW are smaller, shorter and have lower BMI than their peers and thus the glucose load and total dose of glucose is proportionally higher for them. Also subjects born at term but who had been small for their gestational age have been shown in observational studies to present with signs of impaired glucose regulation in adulthood [38], [39]. VLBW subjects as young adults have reduced BMD. This might be due to their smaller size even if the measurements have been corrected for bone size by estimation of volumetric bone density [19], [20]. However, additional adjustment for BMI, lean body mass and fat-% did not significantly alter the results and thus changes were mainly not attributable to body size or body composition. In another study reporting early signs of decreased insulin sensitivity, the body size or composition did not explain the difference in regulation of insulin [19].

Based on the previously available data [40], [41], [42] and the findings in this study, we suggest that the acute changes in glucose homeostasis in humans are mostly regulated by non-skeletal endocrine mechanisms. The difference between the results observed in knockout mice and in humans can potentially be explained additional to the species-specific differences in OC [9] with the role of physiological concentrations of OC or ucOC. While rodent models assess animals with dramatically altered OC levels, clinical research is limited to differences within a normal physiological range. It is unclear whether clinically meaningful changes in markers of bone homeostasis can be observed during an OGTT-induced physiological insulin release. Clamp studies in humans isolating the effect of insulin on bone turnover markers have failed to confirm the effect of physiological changes in insulin on bone metabolism [43], [44]. In a cross-sectional study in an elderly population, OC was significantly correlated with insulin resistance but no association with insulin secretion was observed [12]. The regulatory role of bone metabolism and, in particular, OC on glucose and insulin metabolism may be different in acute and chronic metabolic challenges in humans.

The strengths of this study include the comprehensive and well-described cohort of mostly normoglycemic young adults, with a very limited number of potential confounding factors related to co-morbidities or medications that may influence glycaemia or bone turnover. Further, the subjects were studied close to the age of peak bone mass attainment. Our study also has some limitations. We did not directly measure ucOC but assessed total OC and cOC and estimated the biological activity of OC based on these values. This estimated method may not be sensitive enough to detect small changes in the carboxylation status of OC. Further, we did not have data on vitamin K intake or vitamin K status, which is known to influence carboxylation of osteocalcin [9]. BMD was assessed only in a subset of subjects and even if this portion of subjects is high, it may not represent the entire original cohort of VLBW subjects. However, eligible participants included in BMD analysis or without BMD measurement were similar, as previously described [20]. The criteria used for inclusion of subjects in this study are not entirely the same as those applied when studying glucose regulation [18] because the analysis of OC introduced exclusion of subjects with no information or protocol violation related to the length of fasting period prior to OGTT. Finally, the serum sample volume was insufficient in this cohort for measurements of other bone markers in order to distinguish the putative hormonal role of OC from its role as a marker of bone formation.

In summary, fasting insulin and HOMA-IR index values predicted OC and cOC values in this cohort. This indicates an association between OC and impaired glucose regulation and early signs of peripheral insulin resistance. Our results also suggest that circulating OC or cOC are not involved in the regulation of acute changes in glucose homeostasis. We conclude that serum OC may be involved in the long-term regulation of glucose homeostasis but the role of OC in mediating acute responses to glucose load is not pivotal.
